# Upregulated BAP31 Links to Poor Prognosis and Tumor Immune Microenvironment in Breast Cancer

**DOI:** 10.3390/ijms26135975

**Published:** 2025-06-21

**Authors:** Zhenzhen Hao, Bo Zhao, Xiaoshuang Zhu, Wanting Zhang, Bing Wang

**Affiliations:** Institute of Biochemistry and Molecular Biology, College of Life and Health Sciences, Northeastern University, Shenyang 110819, China; 1710065@stu.neu.edu.cn (Z.H.); 1810068@stu.neu.edu.cn (B.Z.); 2010494@stu.neu.edu.cn (X.Z.); 2110482@stu.neu.edu.cn (W.Z.)

**Keywords:** BAP31, bioinformatics, prognosis, immune infiltration, breast cancer

## Abstract

BAP31, a transmembrane protein in the endoplasmic reticulum, is known for its oncogenic properties, but its role in immunotherapy is not well understood. While BAP31’s involvement in liver, gastric, and cervical cancers has been documented, its role in pan-cancer immune regulation, particularly in breast cancer, remains unexplored. Using TCGA data, analysis via the Xiantao academic and GEPIA2 database showed that BAP31 upregulation correlates with advanced clinical stages and a poor prognosis. ROC analysis demonstrated BAP31’s high accuracy in distinguishing cancerous tissue from normal tissues. Additionally, BAP31 expression is associated with CNV, methylation, TMB, and MSI. In breast cancer, TIMER database analysis revealed that BAP31 expression is inversely correlated with the infiltration levels of myeloid-derived suppressor cells (MDSCs), macrophages, T lymphocytes, B lymphocytes, and neutrophils. Additionally, we investigated the relationship between BAP31 and the expression of major histocompatibility complex (MHC) molecules and chemokine receptors utilizing the TISIDB database. LinkedOmics analysis demonstrated associations between BAP31 and various immune-inflammatory pathways, while also indicating a negative correlation between BAP31 expression and four critical pathways: extracellular matrix receptor interaction, focal adhesion, JAK-STAT signaling, and TGF-β signaling. Furthermore, loss-of-function experiments employing shRNA-mediated knockdown of BAP31 resulted in a marked reduction in cell proliferation and an increase in apoptosis in breast cancer cells, thereby confirming its role in tumor promotion. These findings suggest that BAP31 may serve as a promising prognostic biomarker and a potential target for immunotherapy in breast cancer.

## 1. Introduction

Breast cancer has long been a major global health issue affecting women [1,2]. Despite substantial progress in treatment modalities such as surgery, chemotherapy, and radiotherapy, the 5-year survival rate for patients continues to be suboptimal. Recently, immunotherapy has demonstrated promising results in cancer treatment, notably through the clinical success of immune checkpoint inhibitors, which have significantly improved patient survival rates [3,4,5]. Therefore, it is imperative to explore novel immunotherapy biomarkers or regulatory genes to develop more targeted and efficacious strategies for the treatment of breast cancer.

BAP31 is a 28 kDa ER membrane protein with three transmembrane domains and a cytoplasmic C-terminal tail, encoded on chromosome Xq28 [6,7]. It is crucial for B cell activation [8] and ER-Golgi trafficking [9]. BAP31’s C-terminal can be cleaved by caspase-8 to form the pro-apoptotic fragment P20, influencing apoptosis. It also aids in the transport of proteins like MHC-I, CD11b/CD18, and cytochrome P450 [10,11,12]. Our research shows BAP31 helps degrade CFTRΔF508 by facilitating its transport from the ER to the cytoplasm for proteasomal degradation [13]. In recent years, the role of BAP31 in tumorigenesis has garnered increasing attention. Research indicates that BAP31 is overexpressed across various cancer types and is intricately associated with tumor proliferation, invasion, and metastasis [14,15,16,17]. Furthermore, BAP31 plays a critical role in modulating the activation and function of immune cells, particularly in interactions between T cells and macrophages [18,19]. It influences the M1/M2 polarization of macrophages by inhibiting the activation and differentiation of CD4+ T cells, potentially through the regulation of pivotal molecules such as p-Zap70, p-Lck, and p-Lat within the TCR signaling pathway [18]. Additionally, BAP31 enhances the adhesion and invasion capabilities of tumor cells by promoting EpCAM glycosylation, thereby impacting the immune cells’ recognition and attack on tumors and contributing to immune evasion [20]. However, the applicability of these mechanisms in breast cancer and their effects on the tumor microenvironment remain unexplored.

The tumor microenvironment (TME) consists of various cellular and non-cellular elements, featuring numerous infiltrating immune cells as well as the extracellular matrix [21,22,23]. The complex interactions between immune cells and tumor cells are considered pivotal in the regulation of immune evasion, tumor progression, and therapeutic responses [24,25]. Notably, non-cellular components actively influence the TME’s structure and function through mechanisms such as extracellular matrix remodeling and cytokine network regulation, thereby impacting tumor progression. A comprehensive understanding of the regulatory mechanisms within the TME is crucial for elucidating the fundamental processes of tumorigenesis and tumor development, as well as for devising novel strategies to enhance therapeutic efficacy. Given the significant role of BAP31 in B cell activation, the investigation of its potential associations with the tumor immune microenvironment warrants further exploration.

In the present study, we conducted a comprehensive analysis utilizing multiple databases to examine the elevated expression of BAP31 across various cancers and its correlation with clinical stage and prognosis. In breast cancer, we specifically investigated the relationship between BAP31 expression levels and the tumor microenvironment. Subsequently, we performed an enrichment analysis of genes co-expressed with BAP31. The knockdown of BAP31 resulted in inhibited proliferation and induced apoptosis in breast cancer cells in vitro. Our findings indicate that BAP31 holds potential as a novel prognostic biomarker and therapeutic target for breast cancer patients.

## 2. Results

### 2.1. BAP31 Is Upregulated in a Variety of Human Cancers

**Figure 1:**
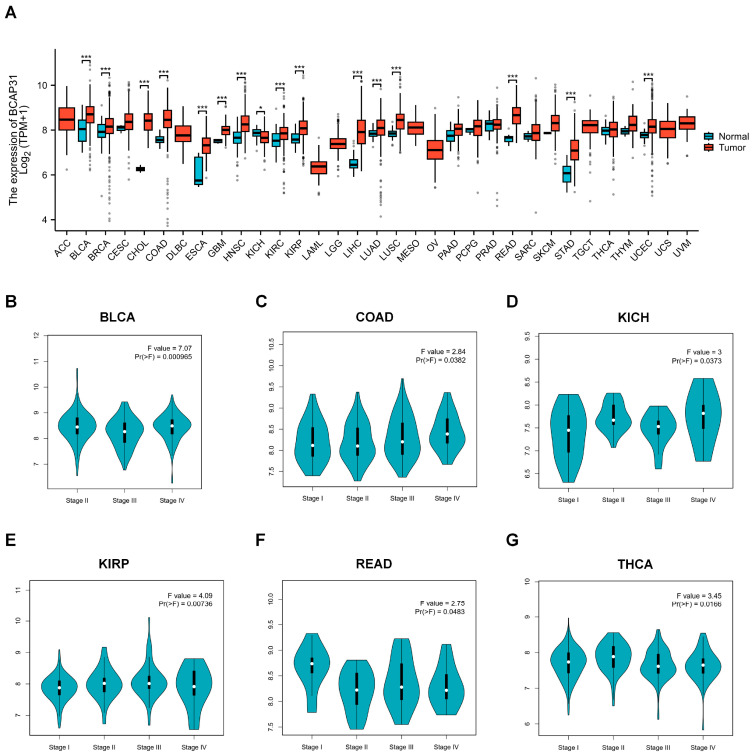
BAP31 expression in multiple human cancers. (**A**) The expression of BAP31 in the Xiantao academic database. (**B**–**G**) The correlation between BAP31 expression and cancer stage. * *p* < 0.05, *** *p* < 0.001.

To initially explore the expression levels of BAP31, we performed an analysis utilizing the Xiantao academic database in conjunction with TCGA datasets. The findings indicated that BAP31 is upregulated in a majority of cancers, including bladder cancer (BLCA), breast cancer (BRCA), cervical cancer (CESC), cholangiocarcinoma (CHOL), colon cancer (COAD), esophageal cancer (ESCA), glioblastoma (GBM), head and neck cancer (HNSC), renal clear cell carcinoma (KIRC), renal papillary cell carcinoma (KIRP), hepatocellular carcinoma (LIHC), lung adenocarcinoma (LUAD), lung squamous cell carcinoma (LUSC), rectal adenocarcinoma (READ), gastric cancer (STAD), and endometrial cancer (UCEC), with statistical significance (*p* < 0.05) (refer to Figure 1A and Table S1). Subsequently, we examined the correlation between BAP31 expression and cancer staging across various cancers using the GEPIA2 platform. Our findings indicate that BAP31 expression is significantly associated with the stages of bladder cancer (BLCA), colon cancer (COAD), renal chromophobe cell carcinoma (KICH), renal papillary cell carcinoma (KIRP), rectal adenocarcinoma (READ), and thyroid cancer (THCA) (see Figure 1B–G).

### 2.2. BAP31 Expression Was Associated with Diagnostic and Prognostic Value in Pan-Cancer

**Figure 2:**
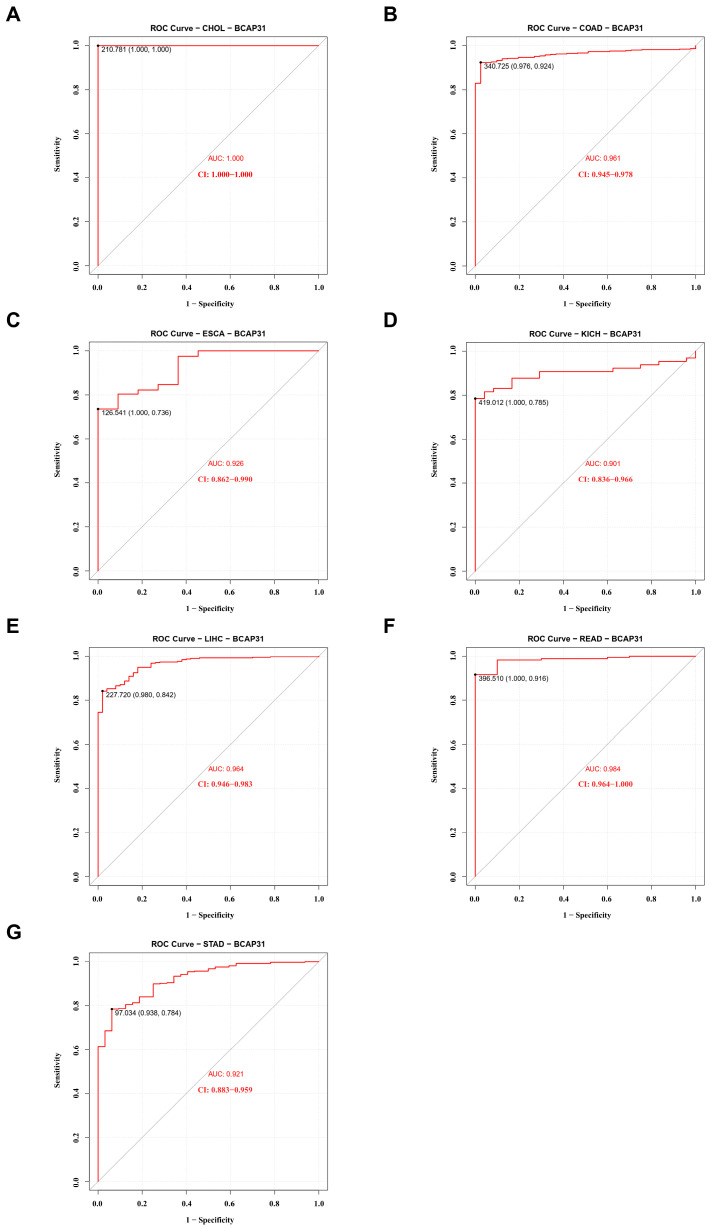
BAP31 expression was associated with diagnostics in pan-cancer. (**A**–**G**) The ROC curves of BAP31 in (**A**) CHOL, (**B**) COAD, (**C**) ESCA, (**D**) KICH, (**E**) LIHC, (**F**) READ, (**G**) STAD.

Subsequently, we evaluated the diagnostic potential of BAP31 across various cancer types utilizing receiver operating characteristic (ROC) curves. As illustrated in Figure 2A–G, BAP31 demonstrated potential as a biomarker for several cancers, including cholangiocarcinoma (CHOL) with an Area Under the Curve (AUC) of 1.000, colon adenocarcinoma (COAD) with an AUC of 0.961, esophageal carcinoma (ESCA) with an AUC of 0.926, kidney chromophobe (KICH) with an AUC of 0.901, liver hepatocellular carcinoma (LIHC) with an AUC of 0.964, rectal adenocarcinoma (READ) with an AUC of 0.984, and gastric cancer (STAD) with an AUC of 0.921.

**Figure 3:**
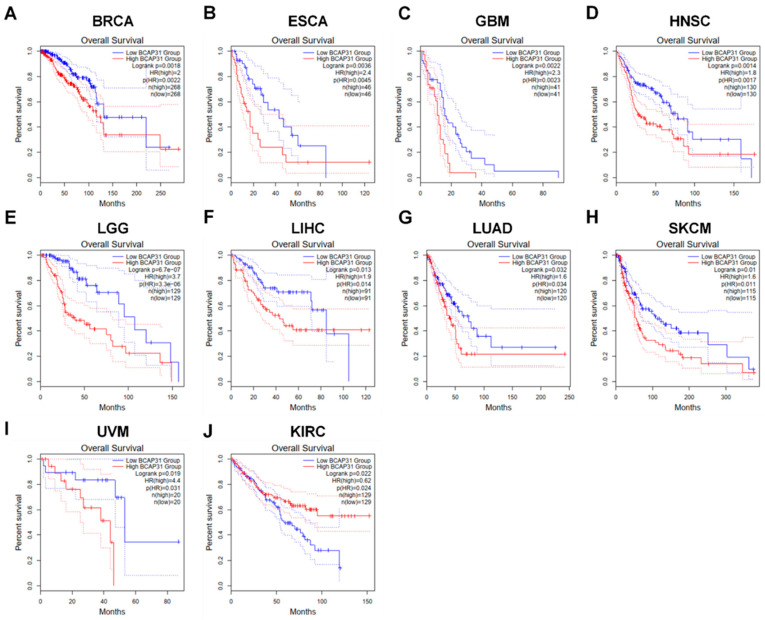
BAP31 expression was associated with prognostic value in pan-cancer. (**A**–**J**) GEPIA2 was used to analyze the relationship between BAP31 expression and OS in (**A**) BRCA, (**B**) ESCA, (**C**) GBM, (**D**) HNSC, (**E**) LGG, (**F**) LIHC, (**G**) LUAD, (**H**) SKCM, (**I**) UVM, (**J**) KIRC.

Furthermore, elevated BAP31 expression was correlated with an unfavorable prognosis. We conducted survival analyses across 33 cancer types, utilizing overall survival (OS) as a metric. Analysis via GEPIA2 revealed that, in breast cancer (BRCA), esophageal cancer (ESCA), glioblastoma multiforme (GBM), head and neck cancer (HNSC), low-grade glioma (LGG), hepatocellular carcinoma (LIHC), lung adenocarcinoma (LUAD), cutaneous melanoma (SKCM), and uveal melanoma (UVM), higher BAP31 expression was associated with reduced OS (Figure 3A–I, *p* < 0.05). Conversely, in papillary renal cell carcinoma (KIRC), increased BAP31 expression was linked to enhanced OS (*p* < 0.05) (Figure 3J). These results indicate that BAP31 expression is significantly associated with tumor prognosis.

### 2.3. Abnormal Expression of BAP31 Was Associated with CNV, Methylation in Pan-Cancer

**Figure 4:**
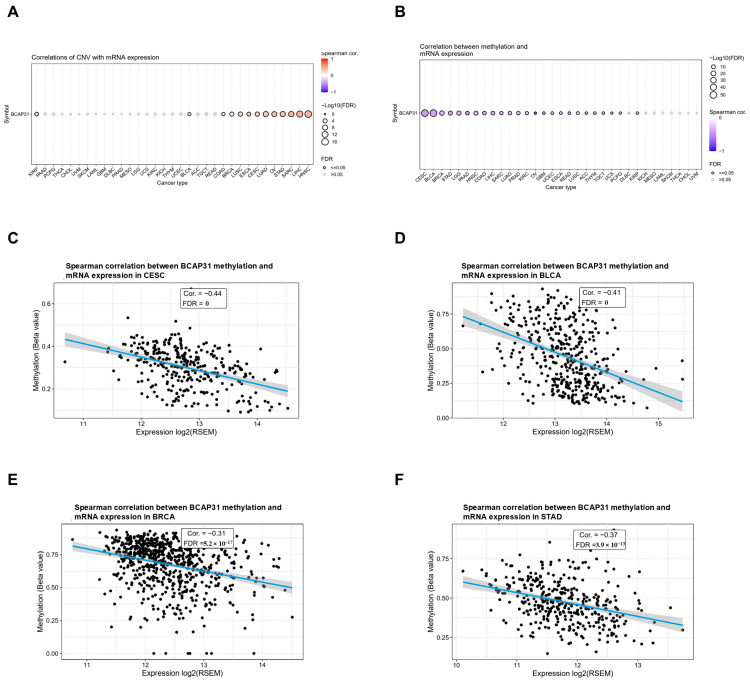
CNV and methylation contribute to driving the abnormal expression of BAP31 in pan-cancers. (**A**) Correlation of CNV and BAP31 mRNA expression in the GSCA database. (**B**) Correlation of methylation and BAP31 mRNA expression in the GSCA database. (**C**–**F**) Correlation of BAP31 methylation and mRNA expression in CESC, BLCA, BRCA, and STAD.

To explore the mechanism of abnormal BAP31 mRNA expression, we first examined the association between gene copy number variation (CNV) and mRNA expression levels. Analysis of data from the GSCA database revealed a significant positive correlation between BAP31 expression and CNV in patients with head and neck cancer (HNSC), liver cancer (LIHC), sarcoma (SARC), and gastric cancer (STAD). Conversely, no significant association was observed in other tumor types (Figure 4A). This indicates that copy number variation (CNV) may not be the sole factor contributing to the aberrant expression of BAP31. Furthermore, the mechanisms underlying its aberrant expression may vary across different tumor types.

DNA methylation serves as an epigenetic mechanism with the potential to substantially influence gene transcription. Our analysis identified a significant inverse correlation between BAP31 mRNA expression and methylation levels across various tumor types. The most pronounced associations were observed in cervical squamous cell carcinoma (CESC), bladder cancer (BLCA), breast cancer (BRCA), and gastric adenocarcinoma (STAD), as depicted in Figure 4B. Specifically, in CESC (r = −0.44, *p* = 0), BLCA (r = −0.41, *p* = 0), BRCA (r = −0.31, *p* = 5.2 × 10^−17^), and STAD (r = −0.37, *p* = 3.9 × 10^−13^), BAP31 methylation exhibited a negative correlation with BAP31 mRNA expression, as illustrated in Figure 4C–F.

### 2.4. Correlation Between BAP31 Expression and Immune Cell Infiltration in Breast Cancer

**Figure 5:**
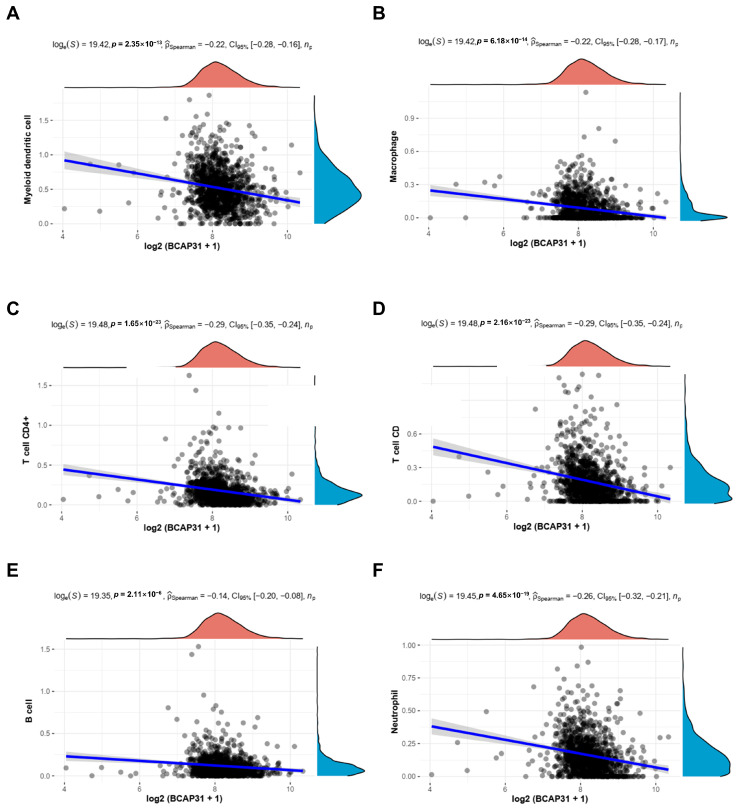
Correlations of BAP31 expression with immune infiltration level in breast cancer. BAP31 expression was negatively correlated with (**A**) myeloid-derived suppressor cells, (**B**) macrophages, (**C**) CD4+ T cells, (**D**) CD8+ T cells, (**E**) B cells, and (**F**) neutrophil infiltration in breast cancer.

The investigation into the association between BAP31 expression and six categories of tumor-infiltrating immune cells was performed utilizing the TIMER database. As depicted in Figure 5A–F, a significant correlation was identified between BAP31 expression and myeloid-derived suppressor cells (r = −0.22, *p* = 2.35 × 10^−13^), macrophages (r = −0.22, *p* = 6.18 × 10^−14^), CD4+ T cells (r = −0.29, *p* = 1.65 × 10^−23^), CD8+ T cells (r = −0.29, *p* = 2.16 × 10^−23^), B cells (r = −0.14, *p* = 2.11 × 10^−6^), and neutrophils (r = −0.26, *p* = 4.65 × 10^−19^).

### 2.5. Correlation Between BAP31 Expression and MHC Molecules in Breast Cancer

**Figure 6:**
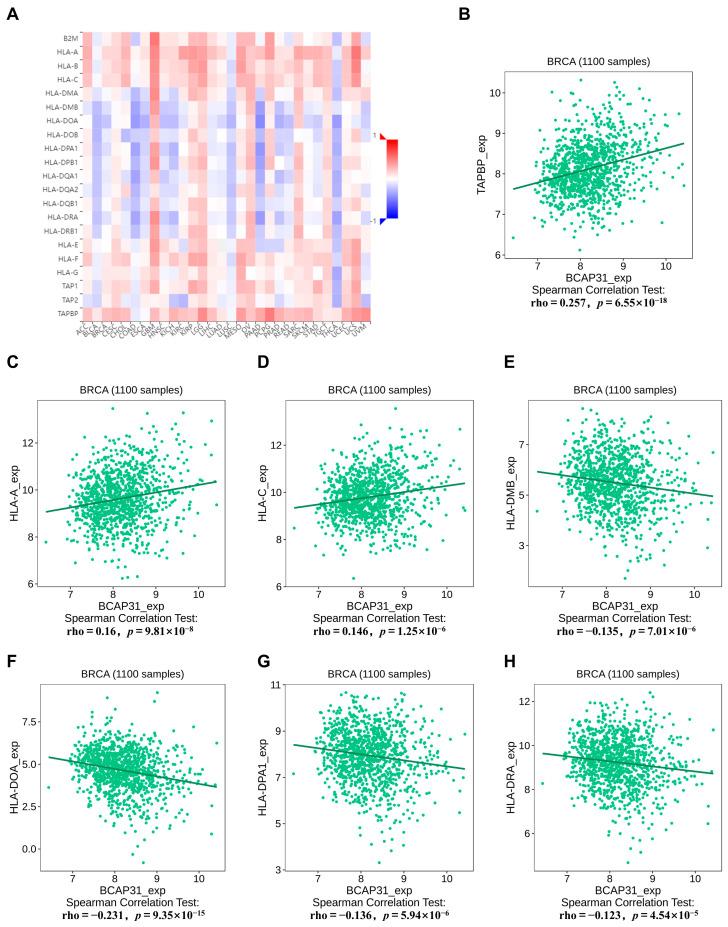
Correlation between BAP31 expression and MHC molecules. (**A**) Relations between the expression of BAP31 and MHC molecules across human cancers. (**B**–**H**) BAP31 was correlated with TAPBP, HLA-A, HLA-C, HLA-DMB, HLA-DOA, HLA-DPA1, HLA-DRA in breast cancer.

The relationship between BAP31 and the major histocompatibility complex (MHC) was examined utilizing the TISIDB database. A noteworthy finding was the significant positive correlation between BAP31 expression and MHCs in glioblastoma multiforme (GBM), contrasted by a negative correlation in thyroid cancer (THCA) (Figure 6A). In the context of breast cancer, BAP31 expression showed a positive correlation with TAPBP (r = 0.257, *p* = 6.55 × 10^−18^), HLA-A (r = 0.16, *p* = 9.81 × 10^−8^), and HLA-C (r = 0.146, *p* = 1.25 × 10^−6^). Conversely, it demonstrated a negative correlation with HLA-DMB (r = −0.135, *p* = 7.01 × 10^−6^), HLA-DOA (r = −0.231, *p* = 9.35 × 10^−15^), HLA-DPA1 (r = −0.136, *p* = 5.94 × 10^−6^), and HLA-DRA (r = −0.123, *p* = 4.54 × 10^−5^) (Figure 6B–H).

### 2.6. Correlation Between BAP31 Expression and Chemokine Receptors in Breast Cancer

**Figure 7:**
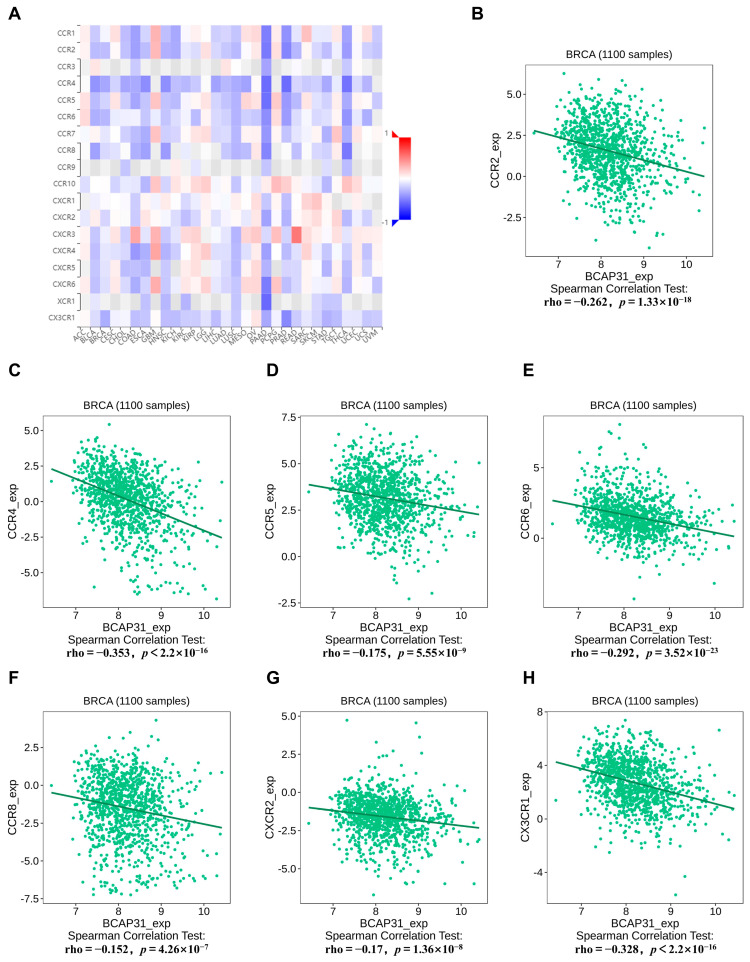
Correlation between BAP31 expression and chemokine receptors. (**A**) Relations between the expression of BAP31 and chemokine receptors across human cancers. (**B**–**H**) BAP31 was correlated with CCR2, CCR4, CCR5, CCR6, CCR8, CXCR2, CX3R1 in breast cancer.

Subsequently, we investigated the association between BAP31 and 18 different chemokine receptors. Notably, an inverse correlation was observed between BAP31 and several chemokine receptors across multiple cancers (Figure 7A). Specifically, in breast cancer, the chemokine receptors that exhibited the most significant correlations were CCR2, CCR4, CCR5, CCR6, CCR8, CXCR2, and CX3CR1 (Figure 7B–H).

### 2.7. Functional Analysis of BAP31 in Breast Cancer

**Figure 8:**
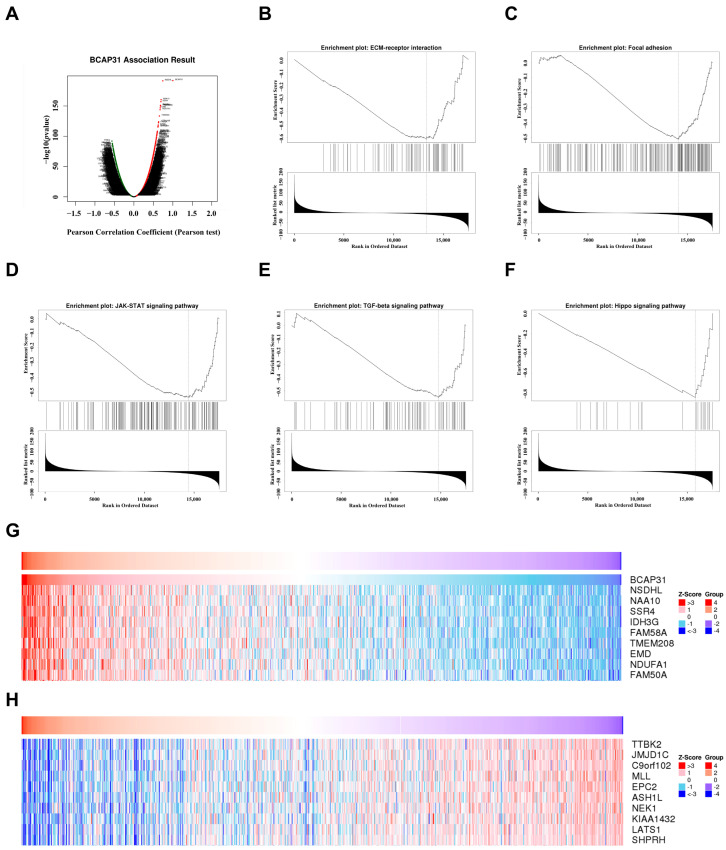
The enrichment analysis of BAP31 co-expression genes in breast cancer. (**A**) The BAP31 co-expression genes in BRCA. KEGG enrichment related signaling pathways: (**B**) extracellular matrix receptor interaction pathway, (**C**) focal adhesion pathway, (**D**) JAK-STAT signaling pathway, (**E**) TGF-β signaling pathway, (**F**) Hippo signaling pathway. (**G**,**H**) The top 10 genes positively and negatively correlated to BAP31.

Subsequently, we utilized the LinkedOmics database to explore the biological role of BAP31 in breast cancer. Figure 8A illustrates the genes exhibiting positive and negative associations with BAP31, while Figure 8G,H present the top 10 genes that are positively or negatively correlated with BAP31. KEGG enrichment analysis revealed that BAP31 is involved in signaling pathways related to the microenvironment, inflammation, and immunity, including the extracellular matrix receptor interaction pathway (Figure 8B), the focal adhesion pathway (Figure 8C), the JAK-STAT signaling pathway (Figure 8D), and the TGF-β signaling pathway (Figure 8E). Notably, these pathways demonstrated an inverse correlation with BAP31. Additionally, a negative correlation was also found between BAP31 and the Hippo pathway, linked to cancer stemness, supporting our earlier findings (Figure 8F). This suggests BAP31 influences tumor growth by affecting cellular stemness and is vital in the immune-inflammatory response in the tumor microenvironment.

### 2.8. Knockdown of BAP31 Inhibits Proliferation and Induces Apoptosis of Breast Cancer Cells

**Figure 9:**
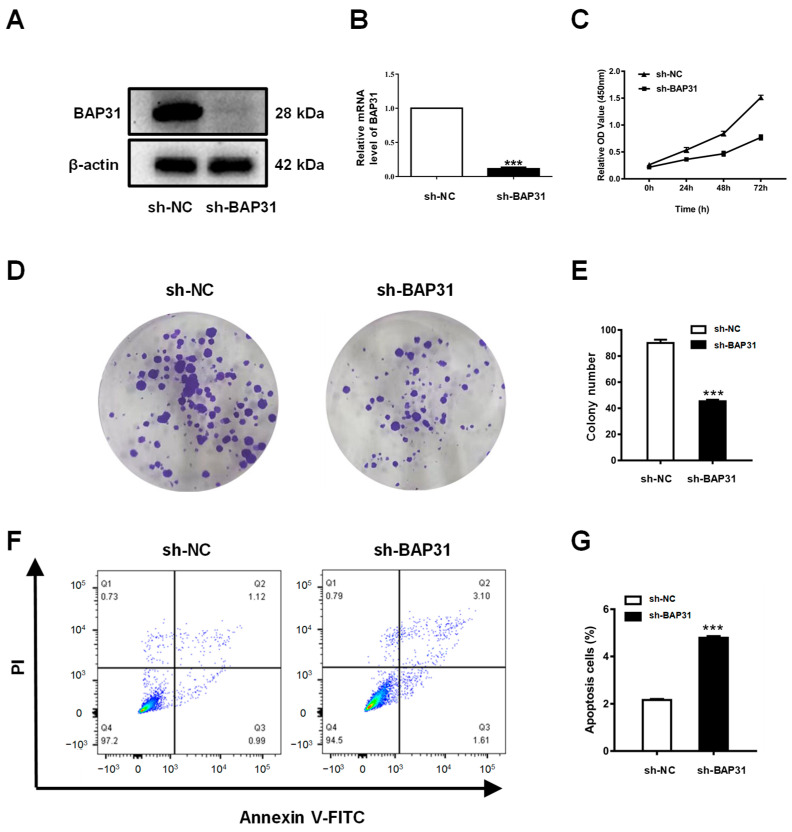
Knockdown of BAP31 suppressed cell proliferation and induced apoptosis in breast cancer cells (**A**) Western blot was used to analyze BAP31 in MCF-7 cells. (**B**) BAP31 relative mRNA expression in MCF-7 cells. (**C**) CCK-8 assay results showing the viability of MCF7 cells. (**D**,**E**) Colony forming ability was determined using the colony formation assay of MCF7 cells. (**F**,**G**) Knockdown of BAP31 induced apoptosis of MCF7 cells. N = 3. *** *p* < 0.001.

In the loss-of-function analysis, MCF7 cells were employed to evaluate the effects of BAP31 knockdown. The MCF7 cell line was transduced with a lentiviral vector encoding a short hairpin RNA (shRNA) targeting BAP31. Both Western blotting and quantitative real-time PCR analyses demonstrated a significant reduction in BAP31 expression in these cell lines (Figure 9A,B). The cell counting kit-8 (CCK8) assay revealed a decrease in the viability of MCF7 cells in vitro following BAP31 knockdown (Figure 9C). This observation was further substantiated by the plate colony formation assay, which showed a marked reduction in the number of cell colonies in the sh-BAP31 group compared with the sh-NC group (depicted in Figure 9D,E). Additionally, the apoptosis assay indicated a significant decrease in apoptosis within the sh-BAP31 group (illustrated in Figure 9F,G). Collectively, these findings suggest that BAP31 may play a crucial role in the progression of breast cancer.

## 3. Discussion

BAP31 was initially identified as a membrane protein with multiple integration sites within the endoplasmic reticulum [26]. Recent research increasingly suggests that elevated BAP31 expression is positively correlated with the development and progression of various cancers. For example, in cervical cancer, BAP31 inhibition has been shown to hinder both disease progression and metastasis [27]. Moreover, BAP31 has been implicated in promoting the migration and invasion of ovarian cancer cells [28]. In colorectal cancer (CRC), BAP31 is overexpressed in tumor tissues, suggesting its potential as a biomarker for CRC [29,30]. Next, we performed a comprehensive bioinformatics analysis of BAP31 using multiple public databases.

In our preliminary analysis, we observed that BAP31 exhibited differential expression levels in the majority of cancerous tissues compared with adjacent normal tissues, suggesting its potential role as an oncogene across various cancer types. This observation is consistent with previous studies reporting the variable expression of BAP31 in multiple cancer forms, including cervical cancer [27], hepatocellular carcinoma [14,31], and lung cancer [32]. Our investigation demonstrated that BAP31 was significantly upregulated and correlated with clinical stages in several cancer types, with receiver operating characteristic (ROC) curve analyses indicating its potential utility as a diagnostic biomarker. Moreover, a comprehensive survival analysis across various cancers revealed that BAP31 expression was significantly associated with prognostic outcomes. Specifically, in breast cancer (BRCA), esophageal cancer (ESCA), glioblastoma (GBM), head and neck cancer (HNSC), low-grade glioma (LGG), liver cancer (LIHC), lung adenocarcinoma (LUAD), cutaneous melanoma (SKCM), and uveal melanoma (UVM), elevated BAP31 levels were correlated with poor overall survival (OS). These findings support prior studies highlighting the impact of BAP31 on survival outcomes in lung and liver cancer. In summary, BAP31 is overexpressed in numerous cancer types and is associated with unfavorable prognoses, indicating its potential utility as a prognostic biomarker in diverse malignancies.

We investigated the link between BAP31 and gene copy number variations (CNVs) and methylation using the GSCA database. The results indicated that BAP31 expression was significantly correlated with CNV in head and neck, liver, sarcoma, and gastric cancers, but not in other cancers, implying that factors beyond CNV contribute to its abnormal expression. Additionally, DNA methylation analysis revealed an inverse relationship between BAP31 mRNA expression and methylation in most tumors, notably in cervical squamous cell carcinoma, bladder, breast, and gastric cancers.

The tumor microenvironment (TME) is acknowledged as a critical factor influencing tumor recurrence and the emergence of drug resistance [33,34]. This milieu is densely populated with a diverse array of immune cells, including myeloid-derived suppressor cells (MDSCs), CD4+ T cells, CD8+ T cells, B cells, and natural killer T (NKT) cells [35,36,37,38]. The presence and functional activity of these immune cells are crucial determinants of the efficacy of immunotherapy interventions. In our study, we identified a significant inverse correlation between the expression of BAP31 and the infiltration levels of various immune cells, such as myeloid-derived suppressor cells, macrophages, CD4+ T cells, CD8+ T cells, B cells, and neutrophils, in breast cancer. Moreover, the expression of BAP31 was found to be inversely correlated with pathways related to inflammation and immune responses in this type of cancer. This correlation implies that BAP31 may have a role in modulating the immune microenvironment of breast tumors, potentially affecting treatment outcomes. Recent studies have underscored the significant association between B cells and T cells and both patient survival and responses to immunotherapy in breast cancer [39,40]. This highlights the role of B cells as active participants in the regulation of tumor dynamics rather than mere passive entities. Furthermore, macrophages, particularly those classified as tumor-associated macrophages (TAMs), have been recognized as essential components of the tumor microenvironment (TME). Research suggests that shifting from a tumor-promoting M2 macrophage phenotype to a tumor-suppressing M1 phenotype may represent a promising strategy to enhance the efficacy of existing cancer therapies, thereby offering new opportunities for improving patient outcomes.

We subsequently investigated the association between BAP31 expression and both major histocompatibility complex (MHC) molecules and chemokine receptors within the context of breast cancer. MHC molecules play a pivotal role in the mechanisms underlying tumor immune evasion. Tumor cells can promote immune evasion by altering the expression of MHC molecules, thereby facilitating tumor progression and evolution. Numerous studies have demonstrated that a decrease in the expression of MHC molecules enables cancer cells to evade detection and elimination by immune cells, thus promoting tumor growth and metastasis, as evidenced in prostate cancer [41] and melanoma [42]. Therefore, examining the role of the MHC in tumorigenesis and progression is of paramount importance. Subsequently, we evaluated the relationship between BAP31 expression and chemokine receptors. In breast cancer, an inverse correlation exists between BAP31 and several chemokine receptors, suggesting that BAP31 may impede the recruitment of immune cells to the tumors. These findings collectively underscore the complex role of BAP31 within the tumor immune microenvironment. However, further investigation is required to elucidate the underlying molecular mechanisms, which will aid in the progression of tumor immunotherapy.

The LinkedOmics database was used to elucidate the biological function of BAP31 in breast cancer. KEGG enrichment analysis demonstrated a negative correlation between BAP31 and several inflammatory and immune-related signaling pathways. Additionally, a negative association was observed between BAP31 and the Hippo pathway, which is linked to cancer stemness, corroborating our previous findings. These results suggest that BAP31 not only influences tumor growth by modulating cell stemness but also plays a significant role in the immune-inflammatory response within the tumor microenvironment. Moreover, the knockdown of BAP31 resulted in decreased proliferation and induced apoptosis in breast cancer cells under laboratory conditions, indicating that BAP31 is crucial for the survival of breast cancer cells. Despite our use of multiple databases, our study faces the limitation of data bias in public datasets. Of note, tumor heterogeneity and treatment exposure may affect BAP31 expression, thereby confounding the findings. Next, more in vitro and in vivo experiments are needed to confirm our findings.

In conclusion, our findings suggest that BAP31 holds potential as a significant prognostic biomarker across diverse cancer types and as a predictive marker for immunotherapy, with particular relevance to breast cancer. These results advance our understanding of the role of BAP31 in tumor immunity and its potential impact on immunotherapeutic approaches.

## 4. Materials and Methods

### 4.1. BAP31 Expression Analysis

The expression levels of the BAP31 gene across various cancer types were obtained from the Xiantao academic database (https://www.xiantao.love; accessed on 17 February 2025). The correlation between BAP31 expression and cancer stage was examined using the “Stage plots” module of GEPIA2 (http://gepia2.cancer-pku.cn/#index; accessed on 17 February 2025). In BAP31 pan-cancer expression analysis, the *t*-test was used according to the data type, and the Benjamini–Hochberg FDR method was used for multiple testing correction (*p* < 0.05). GEPIA2 was used to analyze the association between BAP31 expression and pathological stage using ANOVA, and the Benjamini–Hochberg FDR method was used for multiple test correction (*p* < 0.05). The 33 types of cancer, along with their full names and abbreviations, are detailed in Table S2.

### 4.2. Correlation Between BAP31 Expression and CNV, Methylation

The Gene Set Cancer Analysis (GSCA; http://bioinfo.life.hust.edu.cn/GSCA/#/, accessed on 20 February 2025) database functions as a comprehensive bioinformatics analysis platform, integrating mRNA expression, mutation, immune infiltration, and methylation data derived from TCGA database. The “mutation” module within the GSCA database was utilized to investigate the copy number variations (CNVs) and the methylation status of BAP31, along with their associations with mRNA expression levels. The GSCA platform was used to analyze the association between BAP31 and CNV/methylation using Pearson correlation analysis (CNV) and Spearman rank correlation (methylation), and the Benjamini–Hochberg FDR method was used to correct for multiple testing (significance threshold *p* < 0.05).

### 4.3. Correlation Analysis of BAP31 Expression Levels with Diagnosis and Prognosis in Human Cancers

The diagnostic potential of BAP31 expression in cancer was assessed using receiver operating characteristic (ROC) curve analysis, employing data sourced from TCGA database. An Area Under the Curve (AUC) greater than 0.85 was considered indicative of a high diagnostic value. The association between BAP31 expression and patient prognosis across various cancer types was evaluated using overall survival (OS) metrics. The GEPIA2 platform (http://gepia2.cancer-pku.cn/#index, accessed on 20 February 2025) was employed to investigate cancer prognosis. The log-rank test (survival curve comparison) and the Cox proportional hazards model (HR calculation) were used to analyze the difference in overall survival between BAP31 high/low expression groups. ROC curve analysis of BAP31 was performed using the DeLong test to calculate the significance of the AUC (contrast AUC = 0.5).

### 4.4. Correlation Analysis Between BAP31 and Tumor Microenvironment

The “GENE” module within the TIMER database (http://timer.cistrome.org/, accessed on 20 February 2025) was utilized to evaluate the infiltration levels of immune cells in breast cancer. Furthermore, the TISIDB platform (http://cis.hku.hk/TISIDB/, accessed on 20 February 2025) was employed to investigate the relationship between BAP31 and MHC molecules, as well as chemokine receptors. When BAP31 and the immune cell infiltration levels and immunogenetic molecules were measured, the Spearman rank correlation test was used to evaluate the correlation of expression, and Benjamini–Hochberg FDR correction was performed (*p* < 0.05 was considered significant).

### 4.5. Function and Enrichment Analysis

A gene co-expression analysis of BAP31 was conducted utilizing the LinkedOmics database (https://www.linkedomics.org/login.php, 28 February 2025). This analysis employed the “HiSeq-RNA” platform and concentrated on the “TCGA_BRCA” cohort. The KEGG pathway enrichment was performed via LinkedOmics using the Pearson correlation test with FDR correction (Benjamini–Hochberg). Pathways with *p* < 0.05 and containing five or more overlapping genes were considered significant.

### 4.6. Cell Culture

The MCF7 breast cancer cell line was acquired from the Cell Bank of the Chinese Academy of Sciences (Shanghai, China) and cultured in Minimum Essential Medium (MEM) obtained from Gibco (New York, NY, USA). The culture medium was supplemented with 10% fetal bovine serum (FBS), 1% L-glutamine, and 1% penicillin-streptomycin, all procured from Gibco. The cell cultures were maintained at 37 °C in a CO_2_ incubator provided by Thermo Fisher Scientific (Waltham, MA, USA).

### 4.7. Construction of Stable Cell Lines

The cells underwent a stable knockdown of BAP31 via lentiviral infection, meticulously adhering to established protocols documented in the literature [16]. Specifically, HEK-293T cells were co-transfected with the pLKO.1-puro-shBAP31 vector alongside packaging vectors psPAX2 and pMD2.G. The transfection utilized the Lipo8000 reagent from Beyotime, Shanghai, China. Following a 48 h infection period, the cells were maintained in a puromycin-supplemented medium for one month to ensure the effective selection of successfully transfected cells. All the transfection procedures were conducted in strict accordance with the manufacturer’s recommendations to ensure optimal efficacy and reliability of the experimental outcomes.

### 4.8. RNA Isolation and qPCR

Following cell harvest, BAP31 mRNA levels were quantified using quantitative PCR (qPCR). Sample preparation involved extracting total RNA from the cells utilizing the Total RNA Extraction Kit manufactured by Kangwei, Wuhan, China. Post-extraction, 2 micrograms of RNA were subjected to reverse transcription to generate complementary DNA (cDNA). The subsequent mRNA level analysis was conducted using the UltraSYBR Master Mix from CWBIO, Guangzhou, China. The qPCR primers specific for BAP31 were as follows: forward primer (F)-CCTCTATGCGGAGGTCTTTGT and reverse primer (R)-CCGTCACATCATCATACTTCCGA. For GAPDH, the primers were forward primer (F)-GACAGTCAGCCGCATCTTCT and reverse primer (R)-TTAAAACAGCCCTGGTGAC.

### 4.9. Western Blot Analysis

The cells underwent protein extraction using the RIPA lysis buffer (Beyotime, Shanghai, China). The resultant cell lysates were subjected to separation via SDS-PAGE. Following electrophoresis, proteins were transferred onto a polyvinylidene difluoride (PVDF) membrane, which was subsequently blocked with 5% non-fat milk. The membrane was then incubated with primary and secondary antibodies. Protein detection was performed using Enhanced Chemiluminescence (ECL) buffer (Beyotime), and the signals were captured using a ChemiDoc XRS+ Imaging System (Bio-Rad, Hercules, CA, USA). Quantitative analysis of the protein bands was conducted with Image Lab software (Version 5.2.1). Antibodies specific to BAP31 and β-actin were obtained from Wuhan Sanying Biotechnology (Wuhan, China).

### 4.10. CCK-8 and Colony Formation Assays

Each well of the 96-well plates was seeded with 3000 cells and 100 µL of culture medium. The cells were cultured under standard conditions, and cell viability and proliferation were assessed using a CCK-8 assay from Abbkine Scientific Co., Ltd. (Atlanta, GA, USA) at 24, 48, and 72 h. Optical density at 450 nm was measured with a microplate reader to evaluate cell growth.

Cells were seeded into six-well plates at a density of 2000 cells per well and incubated for 12 days. Post-incubation, the cells were fixed using a 4% formaldehyde solution for 30 min. Subsequently, the cells were stained with a 0.1% crystal violet solution for 15 min to facilitate visualization of the formed colonies. The plates were then rinsed with running water to eliminate excess dye and allowed to air dry at room temperature. For colony count analysis, ImageJ software (version 1.8.0) was utilized to ensure precise and efficient quantification of the colonies.

### 4.11. Cell Apoptosis Assay

Apoptotic activity in the cells was assessed using flow cytometry in conjunction with the Annexin V-FITC Apoptosis Detection Kit (Beyotime). Briefly, the cells were harvested and subjected to multiple washes with cold phosphate-buffered saline (PBS) before being resuspended in 100 µL of 1× binding buffer. Subsequently, the cells were incubated in the dark at room temperature for 15 min with 4 µL of Annexin V-FITC reagent and 8 µL of propidium iodide (PI). Following incubation, 400 µL of 1× binding buffer was added to the stained cells, which were then analyzed using a FACScan flow cytometer (BD Biosciences, San Jose, CA, USA).

### 4.12. Statistical Analysis

The analysis and visualization of the data were conducted utilizing GraphPad Prism 7, in which the results are expressed as mean ± standard deviation (SD) from three separate replicates. For comparative assessments between two groups, Student’s *t*-test was employed, considering a *p*-value lower than 0.05 as indicative of statistical significance.

## Data Availability

The data that support the findings of this study are available from the corresponding author upon reasonable request.

## Supplementary Materials

The following supporting information can be downloaded at https://www.mdpi.com/article/10.3390/ijms26135975/s1.
