# Supplementary material for: Upregulated BAP31 Links to Poor Prognosis and Tumor Immune Microenvironment in Breast Cancer
Source: Int J Mol Sci. 2025 Jun 21;26(13):5975. doi: 10.3390/ijms26135975 (PMC12249588; doi:10.3390/ijms26135975)
Supplement: Supplementary file 1 [file ijms-26-05975-s001.zip › ijms-3691194-supplementary.pdf]

Table S1. The p-values and confidence intervals of human cancers

Group	Confidence intervals (95%CI)	p-values
BLCA	0.4171 – 0.99213	1.28E-05
BRCA	0.15669 – 0.33526	7.94E-08
CESC	-0.20569 – 0.85741	0.1885
CHOL	1.7751 – 2.4394	2.82E-09
COAD	0.76729 – 1.0485	5.85E-21
ESCA	0.80738 – 1.6622	2.38E-06
GBM	0.26426 – 0.68098	0.0009
HNSC	0.5239 – 0.84388	2.39E-15
KICH	-0.31891 – -0.0025186	0.0465
KIRC	0.18958 – 0.40312	1.43E-07
KIRP	0.33347 – 0.62774	4.62E-08
LIHC	1.2192 – 1.5792	1.33E-26
LUAD	0.14692 – 0.35639	4.37E-06
LUSC	0.49167 – 0.70205	5.44E-16
PAAD	-0.24101 – 0.82363	0.2745
PCPG	-0.39091 – 0.59088	0.4107
PRAD	-0.066758 – 0.13788	0.5337
READ	0.75755 – 1.2879	2.89E-07
STAD	0.8696 – 1.3014	2.71E-15
THCA	-0.040725 – 0.17146	0.2303
UCEC	0.27787 – 0.52034	1.07E-08

Table S2. The full names and abbreviations of 33 cancers

abbreviations	full names
ACC	Adrenocortical carcinoma
BLCA	Bladder Urothelial Carcinoma
BRCA	Breast carcinoma
CESC	Cervical squamous cell carcinoma and endocervical adenocarcinoma
CHOL	Cholangiocarcinoma
COAD	Colon adenocarcinoma
COADREAD	Colon adenocarcinoma/Rectum adenocarcinoma Esophageal carcinoma
DLBC	Lymphoid Neoplasm Diffuse Large B-cell Lymphoma
ESCA	Esophageal carcinoma
FPPP	FFPE Pilot Phase II
GBM	Glioblastoma multiforme
GBMLGG	Glioma
HNSC	Head and Neck squamous cell carcinoma
KICH	Kidney Chromophobe
KIPAN	Pan-kidney cohort (KICH+KIRC+KIRP)
KIRC	Kidney renal clear cell carcinoma
KIRP	Kidney renal papillary cell carcinoma
LAML	Acute Myeloid Leukemia

LGG	Brain Lower Grade Glioma
LIHC	Liver hepatocellular carcinoma
LUAD	Lung adenocarcinoma
LUSC	Lung squamous cell carcinoma
MESO	Mesothelioma
OV	Ovarian serous cystadenocarcinoma
PAAD	Pancreatic adenocarcinoma
PCPG	Pheochromocytoma and Paraganglioma
PRAD	Prostate adenocarcinoma
READ	Rectum adenocarcinoma
SARC	Sarcoma
SKCM	Skin Cutaneous Melanoma
STAD	Stomach adenocarcinoma
STES	Stomach and Esophageal carcinoma
TGCT	Testicular Germ Cell Tumors
THCA	Thyroid carcinoma
THYM	Thymoma
UCEC	Uterine Corpus Endometrial Carcinoma
UCS	Uterine Carcinosarcoma
UVM	Uveal Melanoma
